# Neutrophil to lymphocyte ratio, platelet to lymphocyte ratio, and monocyte to lymphocyte ratio in ADHD: a systematic review and meta-analysis

**DOI:** 10.3389/fpsyt.2023.1258868

**Published:** 2023-11-14

**Authors:** Adam Gędek, Szymon Modrzejewski, Marta Gędek, Anna Z. Antosik, Paweł Mierzejewski, Monika Dominiak

**Affiliations:** ^1^Department of Pharmacology, Institute of Psychiatry and Neurology, Warsaw, Poland; ^2^Praski Hospital, Warsaw, Poland; ^3^Faculty of Medicine, Medical University of Lublin, Lublin, Poland; ^4^Chair and Department of Forensic Medicine, Medical University of Lublin, Lublin, Poland; ^5^Independent Public Clinical Hospital No 1, Lublin, Poland; ^6^Department of Psychiatry, Faculty of Medicine, Collegium Medicum, Cardinal Wyszynski University in Warsaw, Warsaw, Poland

**Keywords:** attention-deficit hyperactivity disorder, neutrophil-to-lymphocyte ratio, platelet-to lymphocyte ratio, monocyte-to lymphocyte ratio, inflammation, blood parameters

## Abstract

**Introduction:**

Attention-deficit hyperactivity disorder (ADHD) is a neurodevelopmental disorder with an unclear etiology. Systemic inflammation and immune dysregulation may play a role in the pathogenesis of ADHD. Morphology-derived parameters such as neutrophil to lymphocyte ratio (NLR), platelet to lymphocyte ratio (PLR), and monocyte to lymphocyte ratio (MLR), have been proposed as peripheral biomarkers of the immune-inflammatory process in various diseases. However, studies examining their role in ADHD remain inconclusive.

**Methods:**

A systematic review and a meta-analysis were conducted to evaluate the association between NLR, MLR, PLR and ADHD. Relevant articles were identified, screened, and assessed for quality according to PRISMA guidelines. Moreover, a qualitative and quantitative analyses were performed.

**Results:**

The review contained eight eligible studies, five of which were included in the meta-analysis. The meta-analysis showed that ADHD patients had higher NLR and PLR values compared to health controls. No significant difference in MLR value was observed between the two groups. Analysis in relation to ADHD subtypes showed no significant differences in inflammatory markers in any of the included studies as well. The influence of medical treatment on these ratios could not be adequately assessed due to limited data.

**Conclusion:**

ADHD patients exhibit higher NLR and PLR than healthy controls, which may indicate the potential immune-inflammatory involvement in this disorder. Further studies on inflammatory markers and ADHD, especially those considering the impact of treatment and clinical symptoms, are essential to comprehensively understand this association.

## 1. Introduction

Attention-deficit hyperactivity disorder (ADHD) is a neurodevelopmental disorder characterized by persistent patterns of inattention, hyperactivity, and impulsivity (1). The disorder affects individuals across different age groups, carrying significant implications for daily functioning and the overall quality of life for them and their families (2). Despite its prevalence and impact, the exact underlying pathophysiological mechanisms remain undiscovered (3).

In recent years, there has been a growing interest in exploring the role of systemic inflammation and immune dysregulation in the pathogenesis of ADHD (4, 5). Inflammatory and autoimmune diseases, such as eczema, asthma, rheumatoid arthritis, type 1 diabetes, or hypothyroidism, co-occur significantly more frequently with ADHD (6, 7). Studies to date have suggested that patients with ADHD have higher levels of inflammatory cytokines, such as Il-6 or Il-10, than healthy individuals (8, 9). Furthermore, some of the studies have also demonstrated an association of inflammatory markers with disease severity (10). In addition, it has been observed that the patients who take psychostimulants display lower levels of inflammatory markers (11). Moreover, several lines of evidence from genetic studies have indicated links between ADHD and polymorphisms in genes associated with inflammatory pathways (5).

Alterations in various peripheral blood cell ratios, such as neutrophil to lymphocyte ratio (NLR), platelet to lymphocyte ratio (PLR), and monocyte to lymphocyte ratio (MLR), have been shown to serve as potential biomarkers of immune-inflammatory processes or poor prognoses in various diseases, such as cancers, cerebrovascular, cardiovascular, or autoimmune diseases (12–16). In recent years, the assessment of NLR, MLR, and PLR in patients with mental illnesses has also been investigated. These inflammatory markers were elevated in depressed, bipolar, or non-affective psychosis patients (17–20).

To date, several studies have investigated the link between NLR, PLR, MLR, and ADHD, but the findings have been inconsistent. Some studies have reported elevated ratios among individuals with ADHD, suggesting a potential role of inflammation in the pathophysiology of the disorder (21, 22). However, other studies have found no significant differences in these parameters among ADHD patients, pointing to the complexity of the immune-inflammatory mechanisms involved (23, 24).

Therefore, the aim of this study was to conduct a systematic review and meta-analysis to evaluate the association between immune-inflammatory markers such as NLR, PLR, MLR, and ADHD. Specifically, we planned to: (1) compare NLR, MLR, PLR levels between ADHD patients and healthy controls; (2) assess the effects of psychostimulant treatment on NLR, MLR, PLR in ADHD patients; (3) evaluate the association of NLR, MLR, PLR levels with ADHD subtypes; (4) evaluate the association of NLR, MLR, PLR levels with ADHD symptoms and their severity. To our best knowledge, no systematic review on this topic has been conducted to date.

We hypothesized that NLR, MLR and PLR levels differ significantly between ADHD patients and healthy controls, and that psychostimulant treatment affects these markers in ADHD subjects. We also hypothesized that NLR, MLR and PLR level differ in various subtypes of ADHD and are related to symptoms and severity of the disease. This systematic review and meta-analysis provide a comprehensive insight into the relationship between NLR, MLR, PLR and ADHD.

## 2. Materials and methods

This systematic review was conducted according to the PRISMA statement (Preferred reporting items for systematic review and meta-analysis) (25).

### 2.1. Search strategy

A literature search in April 2023 included the contents of Pubmed, Scopus, and Web of Science electronic databases without any filters. Additionally, clinicaltrialregistry.gov was searched. The following search strategy was used: (“neutrophil-to-lymphocyte ratio” OR “NLR” OR “neutrophil to lymphocyte ratio” OR “neutrophil/lymphocyte ratio” OR “neutrophil-lymphocyte ratio” OR “neutrophil lymphocyte ratio” OR “platelet-to-lymphocyte ratio” OR “PLR” OR “platelet to lymphocyte ratio” OR “platelet/lymphocyte ratio” OR “platelet-lymphocyte ratio” OR “platelet lymphocyte ratio” OR “monocyte-to-lymphocyte ratio” OR “MLR” OR “monocyte to lymphocyte ratio” OR “monocyte/lymphocyte ratio” OR “monocyte-lymphocyte ratio” OR “monocyte lymphocyte ratio”) AND (“ADHD” OR “Attention Deficit Hyperactivity Disorder”). In addition, references from selected articles were screened to confirm potentially related studies.

### 2.2. Inclusion and exclusion criteria

The following criteria were a condition for the inclusion of the studies in this systematic review: (1) observational study design (case-control, cohort, or cross-sectional); (2) concerning ADHD patients over 6 years of age and under 18 years of age and controls without mental disorders; (3) at least one of the NLR, MLR, or PLR levels were measured; (4) published in English. The exclusion criteria were as follows: (1) reviews, letters, and conference abstracts; (2) non-human studies; (3) duplicate data; (4) full text was not available; (5) not in English. Two investigators worked independently to complete the preliminary screening through browsing titles and abstracts. The final decisions were made after reviewing the full texts. Disagreements between the researchers were resolved by consultation with the third author.

### 2.3. Data extraction

The original data was extracted from the included studies by two of the researchers. The following information was collected: the surname of the first author, publication year, country, matching information for sex and age, diagnostic criteria, sample size, mean age, percentage of men, information about comorbidities, medical treatment and subtypes of ADHD, peripheral blood cell ratios (NLR, MLR, PLR). Any divergences were resolved through discussion between the two investigators and consultation by a third researcher.

### 2.4. Quality assessment

The quality of the selected studies was independently evaluated by two authors with the Newcastle-Ottawa scale (NOS) (26). The study was assessed with respect to three aspects: Selection, Comparability, and Exposure. A maximum of one star was awarded in each category for Selection and Exposure, while a maximum of two stars could be awarded for Comparability. Studies were rated from 0 to 9, with those scoring from 0 to 2 being ranked as poor quality, 3 to 5 as fair quality and 6 to 9 as high quality. Any disagreements were managed by group discussion. Only high and fair quality studies were included in meta-analysis.

### 2.5. Statistical analysis

The meta-analysis was conducted using RevMan5 (version 5.4; Cochrane Collaboration) software. Continuous outcomes were pooled as standardized mean difference (SMD). Heterogeneity was evaluated visually on the Forest plot and statistically using the Chi^2^, I^2^, and Tau^2^. Thresholds from Cochrane Collaboration were consistent with interpretation of heterogeneity: 0–40% might be not important; 30–60% may represent moderate heterogeneity; 50–90% may represent substantial heterogeneity, and 75–100% high level of heterogeneity. A fixed-effect model was used to analyze and *p* < 0.05 was set as a statistical significance. The subgroup analysis with regard to the psychostimulant treatment used was planned.

## 3. Results

### 3.1. Study selection

**FIGURE 1:**
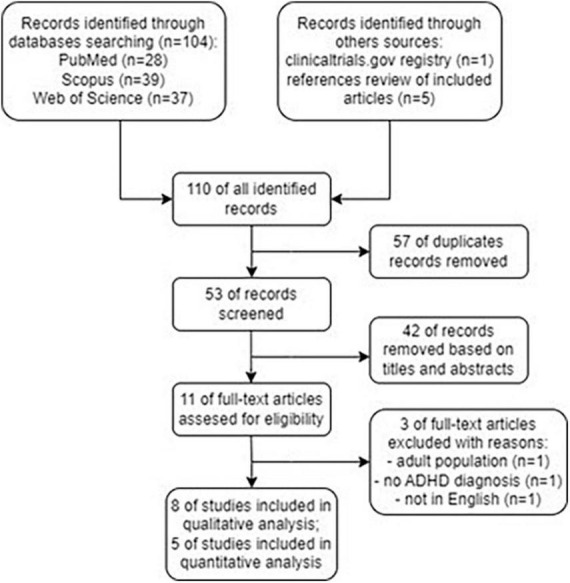
Flowchart showing an overview of the study selection process.

A total of 110 articles were identified through the search strategy. After removing 57 duplicates, 53 remained for titles and abstracts screening. Initially, eleven full-text articles were carefully assessed for eligibility. However, this number was later reduced to eight eligible articles, which were used for data extraction and summarization of the results (21–24, 27–30). Five of the articles were included in quantitative analysis (21–24, 30). The search process flow and results are detailed in Figure 1.

### 3.2. Characteristics of included studies

Among included studies 8, 7, and 6 of them investigated the NLR, PLR, and MLR, respectively. The papers were published between 2018 and 2022. All studies included both male and female subjects. The age of patients ranged from 8.3 ± 1.7 to 10.33 ± 3.15 years old. Seven of these studies were conducted in Turkey, one in Egypt. In all papers ADHD diagnoses were identified based on DSM-V criteria, while in one study additionally with the Schedule for Affective Disorders and Schizophrenia for School-Age Children – Present and Lifetime Version (K-SADS-PL) (23). Patients with other mental disorder were excluded in all cases. All ADHD children were diagnosed with delayed language development (DLD) in one of the studies (27) and in another with specific learning disorder (SLDs) (30). One study excluded patients with other psychiatric disorders, with the exception of oppositional defiant disorder (ODD) (24). In all but one study, participants received no treatment, in one study the majority of patients (85%) were treated. One study did not provide information about diagnosis criteria, numbers and sex of participants, comorbidities and medical treatment (29). The detailed information about included studies are presented in Table 1.

**TABLE 1:** Characteristics of included studies.

References Country	Diagnosis criteria	Matching age and sex	Individuals with ADHD			Controls			Main findings	Comorbidities	Medical treatment	Association with subtypes of ADHD and correlation with symptoms/severity of disease
			N	Mean age	Males (n)	N	Mean age	Males (n)				
Onder et al. (21)Turkey	DSM-V	Yes	100	9.76 ± 2.65	83	99	10.21 ± 2.74	82	The mean NLR, PLR were significantly higher in ADHD patients group than in HC (both *p* < 0.001).	Organic mental syndromes, psychotic disorders, mental retardation, autism spectrum disorders, mood and anxiety disorders, and also systemic diseases (e.g., malignancy, cardiac disease, hematologic disease) that could lead to abnormal hemogram results were in the exclusion criteria.	Of the 100 patients in the ADHD group, 85 (85%) patients used at least one medication (methylphenidate or atomoxetine), and 15 (15%) patients received no psychopharmacologic treatment. The most commonly used drug was methylphenidate (*n* = 76), and atomoxetine (*n* = 14). In addition, 19 patients were receiving antipsychotic treatment. In the ADHD group, no significant difference was determined between patients who received psychopharmacological treatment for ADHD and patient that did not with regard to NLR and PLR. No significant correlation was found between the duration of medication use and NLR and PLR in the group using atomoxetine and/or methylphenidate.	There were no significant differences in NLR and PLR values among the subtypes of ADHD. No associations were found between NLR and PLR and T-DSM-IV-S scores.
Topal et al. (23)Turkey	K-SADS-PL, DSM-V	Yes	61	8.7 ± 1.5	46	70	8.4 ± 3.8	49	Only the mean NLR differed significantly across diagnostic groups (ADHD, ASD, controls) in univariate ANOVAs (*p* = 0.016 for NLR; *p* = 0.896 for PLR; *p* = 0.343 for MLR)	Comorbid chronic medical diseases, those with infectious diseases during the application and children refusing participation were in the exclusion criteria.	Use of long-term medications and vitamin supplements was in the exclusion criteria.	NLR tended to correlate with CD (Conduct Disorder) scores on T-DSM-IV-S, although not reaching significance.
Fahiem and Mekkawy (27)Egypt	DSM-V	Yes	70	8.7 ± 1.9	49	44	8.3 ± 1.7	30	NLR, MLR, and PLR were significantly higher in the ADHD group than HC (*p* < 0.001 for NLR; *p* < 0.001 for PLR; *p* = 0.002 for MLR)	Only patients with ADHD and DLD were included in this study. Epilepsy, mental sub-normality, autism, organic brain lesions, psychiatric conditions, hematological disease, acute/chronic medical illnesses were in the exclusion criteria.	Only patients, who did not receive any medications were included in the study. Drug history was in the exclusion criteria.	The NLR, PLR, and MLR levels in the ADHD subtypes of children were non-significant.
Avcil (28)Turkey	DSM-V	Yes	82	8.9 ± 1.96	65	70	9.2 ± 2.03	60	NLR, PLR, and MLR were significantly higher in children with ADHD than in the HC (*p* < 0.001, *p* = 0.006, *p* = 0.023 respectively)	Seizure disorders, mental retardation, autistic disorder, organic brain damage, psychotic disorder, conduct disorder, elevated blood pressure, hypercholesterolemia, any other acute or chronic physical illnesses, or smoking were in the exclusion criteria.	Only drug-naive participants were included in the study. A history of any drug use during the last month was in the exclusion criteria.	No associations were found between the inflammatory markers and questionnaire scores (T-DSM-IV-S subscale and CTRS scores).
Akinci et al. (22)Turkey	DSM-V	Yes	347	9.68 ± 2.63	264	205	9.69 ± 2.56	150	The mean NLR, and PLR were significantly higher in patients with ADHD than in HC (*p* = 0.002, *p* = 0.014 respectively); the mean MLR was not significantly different (*p* = 0.663)	No other psychiatric disorder was in the inclusion criteria. Concurrent psychiatric diagnoses, presence of an autoimmune disease, acute infection, systemic disease, epilepsy, allergic diseases, as well as other psychiatric, hematologic, endocrine, immunological, renal diseases, neurological and cardiological diseases were in the exclusion criteria.	No psychiatric medication history at any period of life was in the inclusion criteria. Use of all types of drugs was in the exclusion criteria.	There were no significant differences in NLR, PLR, and MLR values among the subtypes of ADHD.
Alpay et al. (29)Turkey	No info	No info	30	No info	No info	30	No info	No info	The mean NLR values were significantly higher in patients with ADHD than in HC (*p* < 0.001)	No info	No info	No info
Yektas et al. (30)Turkey	DSM-IV	Yes	80	9.1 ± 1.6*	68*	75	9.3 ± 1.5	50	NLR, MLR, PLR were as follows in ADHD ad HC groups: NLR 1.8 (SD 1.2) vs 1.3 (SD 0.4); MLR 0.2 (SD 0.1) vs. 0.2 (SD 0.1); PLR 124.4 (47.9) vs 111.0 (23.7)	Only patients with Specific learning disorders (SLDs) and ADHD were included in the study. Children with mental retardation/intellectual disabilities, autistic spectrum disorders, organic brain damage, epilepsy, psychotic disorders, and any other acute or chronic physical illnesses were excluded from the study.	Only drug-naive participants were included in the study.	Parent rated hyperactive/impulsive scores correlated significantly with PLR (rho = 0.30, *p* = 0.002). Parent rated inattention, oppositional and conduct disorder symptoms did not correlate significantly with any of the peripheral inflammatory markers. Teacher rated hyperactivity/impulsivity correlated significantly with PLR (rho = 0.21, *p* = 0.036). Teacher rated oppositional behaviors correlated significantly with PLR (rho = 0.40, *p* = 0.000). Teacher ratings of conduct disorder symptoms correlated significantly with PLR (rho = 0.29, *p* = 0.003).
Aksu and Dağ (24)Turkey	DSM-V	Yes	169	9.68 ± 2.98	130	59	10.33 ± 3.15	48	NLR, PLR, MLR values did not differ between the groups.	Comorbid psychiatric disorders were screened and ODD was included, all other psychiatric disorders were excluded. Exclusion criteria also contained an intellectual disability and/or autism spectrum disorder according to DSM-5 criteria, an acute, chronic or inflammatory disease.	Use of psychotropic medication was in the exclusion criteria.	There were no significant differences in NLR, PLR, and MLR values among the subtypes of ADHD.

HC, health controls; T-DSM- IV-S, the Turgay DSM-IV-Based Child and Adolescent Behavior Disorders Screening and Rating Scale; CTRS, The Conners’ Teacher Rating Scale; ODD, oppositional defiant disorder; SLDs, specific learning disorders. *Data for the entire study group including patients with ADHD and SLD (n = 80) and pure (n = 20); no separate data for ADHD and SLD patients.

### 3.3. Quality assessment of included studies

Table 2 provides quality scores for the papers, assessing risk of bias. Six of the studies were of high quality, one was of fair quality (30), and one was of poor quality (29). Only the high and fair quality studies were included in meta-analysis.

**TABLE 2:** Quality assessment of included studies with Newcastle-Ottawa Scale (NOS).

Studies	Selection				Comparibility	Exposure			Total
	**Is the case definition adequate?**	**Representativeness of the cases**	**Selection of controls**	**Definition of controls**	**Comparability of cases and controls on the basis of the design or analysis**	**Assessment of exposure**	**Same method of ascertainment for cases and controls**	**Non-response rate**	
Onder et al. (21)	1	1	0	1	2	1	1	0	7
Topal et al. (23)	1	1	1	1	2	1	1	0	8
Fahiem and Mekkawy (27)	1	1	1	1	2	1	1	0	8
Avcil (28)	1	1	1	1	2	1	1	0	8
Aksu and Dağ (24)	1	1	0	1	2	1	1	0	7
Akinci et al. (22)	1	1	0	1	2	1	1	0	7
Alpay et al. (29)	0	1	0	1	0	0	0	0	2
Yektas et al. (30)	1	1	0	1	0	1	1	0	5

### 3.4. Patients with ADHD vs. healthy controls

Table 3 provides information about inflammatory markers (NLR, MLR, PLR) in studies included in the meta-analysis.

**TABLE 3:** Studies included in meta-analysis and inflammatory markers ratio.

Inflammatory ratio	References	Mean value in patients with ADHD/sample size		Mean value in controls/sample size		Medical treatment (yes/no)	Psychiatric comorbidities (yes/no)
NLR	Akinci et al. (22)	1.59 ± 0.84	347	1.42 ± 0.74	205	no	no
	Onder et al. (21)	1.74 ± 0.71	100	1.17 ± 0.49	99	yes	no
	Topal et al. (23)	1.47 ± 0.77	70	1.02 ± 0.33	61	no	no
	Yektas et al. (30)	1.8 ± 1.2	80	1.3 ± 0.4	75	no	no, except SLDs
	Aksu and Dağ (24)	1.39 ± 0.75	169	1.38 ± 0.65	59	no	no, except ODD
PLR	Akinci et al. (22)	114.26 ± 37.01	347	110.12 ± 44.66	205	no	no
	Onder et al. (21)	140.32 ± 45.70	100	108.29 ± 31.57	99	yes	no
	Topal et al. (23)	120.98 ± 38.02	70	109.10 ± 29.61	61	no	no
	Yektas et al. (30)	124.4 ± 47.9	80	111.0 ± 23.7	75	no	no, except SLDs
	Aksu and Dağ (24)	117.06 ± 34.40	169	118.19 ± 45.85	59	no	no, except ODD
MLR	Akinci et al. (22)	0.19 ± 0.08	347	0.18 ± 0.09	205	no	no
	Topal et al. (23)	0.21 ± 007	70	0.17 ± 0.04	61	no	no
	Yektas et al. (30)	0.2 ± 0.1	80	0.2 ± 0.1	75	no	no, except SLDs
	Aksu and Dağ (24)	0.22 ± 0.09	169	0.21 ± 0.10	59	no	no, except ODD

ODD, oppositional defiant disorder; SLDs, specific learning disorders.

#### 3.4.1. NLR

NLR in ADHD patients was evaluated in eight studies. The mean NLR were significantly higher in ADHD patients group than in healthy controls in six of them (6/8, 75%). the difference between groups was not significant in one of the studies (24), in another one the statistical significance was not reported (30).

**FIGURE 2:**
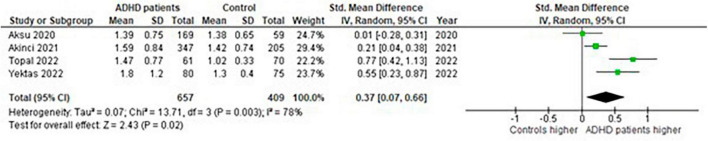
Meta-analysis forest plot for NLR.

Three of the eight studies were excluded from the meta-analysis: two due lack of raw data (27, 28) and one due to poor quality (29). The pooled analyses showed that the ADHD subjects had higher levels of NLR than the healthy controls. The heterogeneity of the studies was substantial (I^2^ = 86%, Chi^2^ = 28.75, df = 4, Tau^2^ = 0.12, *p* < 0.00001). The standardized mean difference (SMD) was 0.49 [0.15, 0.82]. Test for overall effect: *Z* = 2.87 (*p* = 0.004). The analysis found discrepancies reflected in the considerable heterogeneity of analyzed studies. Specifically, we identified one significant outlier (21). This study was the only one in which the majority of the patients (85%) received medical treatment. Thus, we performed a sensitive analysis excluding this study. It resulted in a substantial decrease in heterogeneity (I^2^ = 78%, Chi^2^ = 13.71, df = 3, Tau^2^ = 0.07, *p* = 0.003). The result of meta-analysis did not change substantially. SMD was 0.37 [0.07, 0.66], test for overall effect: *Z* = 2.43, *p* = 0.02 (Figure 2).

#### 3.4.2. PLR

PLR in ADHD patients was evaluated in seven studies. The mean PLR were significantly higher in ADHD patients group than in healthy controls in four of them (4/7, 57%). The difference between groups was not significant in two of the studies (23, 24), in another one the statistical significance was not reported (30).

**FIGURE 3:**
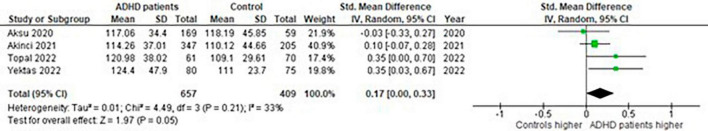
Meta-analysis forest plot for PLR.

Two of seven studies were excluded from the meta-analysis due to lack of raw data (27, 28). The heterogeneity of included studies was substantial (I^2^ = 81%, Chi^2^ = 21.36, df = 4, Tau^2^ = 0.08, *p* = 0.0003). The standardized mean difference (SMD) was 0.31 [0.03, 0.59]. Test for overall effect: *Z* = 2.14 (*p* = 0.03). The sensitivity analysis was performed and one significant outlier was identified (21). This study was the only one in which the majority of patients (85%) received medical treatment. Excluding this one outlier resulted in a substantial decrease in heterogeneity (I^2^ = 33%, Chi^2^ = 4.49, df = 3, Tau^2^ = 0.01, *p* = 0.21). SMD was 0.17 [0.00, 0.33], test for overall effect: *Z* = 1.97, *p* = 0.05 (Figure 3).

#### 3.4.3. MLR

MLR in ADHD patients was evaluated in six studies. The mean MLR were significantly higher in ADHD patients group than in healthy controls in two of the studies (2/6, 33%) (27, 28). The difference between groups was not significant in three of the studies (22–24), in another one the statistical significance was not reported (30).

**FIGURE 4:**
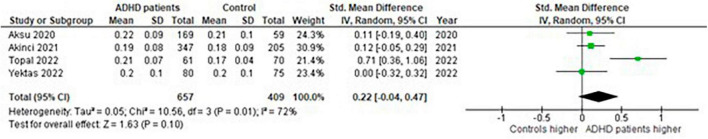
Meta-analysis forest plot for MLR.

Two of six studies were excluded from the meta-analysis due to lack of raw data (27, 28). The heterogeneity was substantial (I^2^ = 72%, Chi^2^ = 10.56, df = 3, Tau^2^ = 0.05, *p* = 0.01). The standardized mean difference (SMD) was 0.22 [−0.04, 0.47]. Test for overall effect: *Z* = 1.63 (*p* = 0.10) (Figure 4).

### 3.5. The association of NLR, PLR, and MLR with regard to the subtypes of ADHD

Four of the studies evaluated these inflammatory markers in ADHD subtypes (21, 22, 24, 27). Two of the studies did not provide any raw data on inflammatory parameter values (21, 27) and one did not report the exact number of patients for each disease subtype (24). Therefore, a meta-analysis was not feasible. However, the narrative synthesis appears to provide consistent results.

The mean NLR and PLR values with regard to the subtypes of ADHD were assessed in four of the studies. There were no statistically significant differences in these values among the subtypes of ADHD in all of them (4/4, 100%) (21, 22, 24, 27).

The mean MLR value in context of ADHD subtypes was assessed in three of the studies. There was no statistically significant difference in MLR value among the subtypes of ADHD in three of them (3/3, 100%) (22, 24, 27).

### 3.6. The influence of medical treatment on NLR, PLR, and MLR

Only one study assessed patients with ADHD on medical treatment (85% participants) (21). This study was also a cause of heterogeneity in the meta-analysis. However, in the ADHD group, no significant difference was observed between patients who received psychopharmacological treatment for ADHD and patients who did not, with regard to NLR and PLR. Moreover, no significant correlation was found between the duration of medication use and the values of NLR or PLR in the group using atomoxetine and/or methylphenidate (21).

### 3.7. The correlation of NLR, PLR and MLR with disease symptoms and severity

The correlation between inflammatory markers and scores on clinical scales was assessed in three studies. One of the studies showed no associations between NLR and PLR and T-DSM-IV-S scores (Turgay DSM-IV-Based Child and Adolescent Behavior Disorders Screening and Rating Scale) (21). Another one found no associations between the inflammatory markers (NLR, MLR, PLR) and questionnaire scores (T-DSM-IV-S subscale and CTRS scores - The Conners’ Teacher Rating Scale) (28). In third one NLR tended to correlate with CD (Conduct Disorder) scores on T-DSM-IV-S, although not reaching significance (23).

One study assessed correlation between inflammatory markers and parent/teacher-rated symptoms on T-DSM-IV-S (30). Parent-rated hyperactive/impulsive scores correlated significantly with PLR (rho = 0.30, *p* = 0.002). Nevertheless, parent-rated inattention, oppositional and conduct disorder symptoms did not correlate significantly with any of the peripheral inflammatory markers. Teacher-rated hyperactivity/impulsivity, oppositional behaviors and conduct disorder symptoms correlated significantly with PLR (rho = 0.21, *p* = 0.036; rho = 0.40, *p* = 0.000; rho = 0.29, *p* = 0.003, respectively) (30).

## 4. Discussion

The present study aimed to systematically review and meta-analyze data on the association between - morphology-derived parameters - NLR, PLR, and MLR values and ADHD. To the best of our knowledge, this is the first systematic review and meta-analysis examining the above-mentioned associations in ADHD.

This study demonstrated that NLR and PLR values are higher in ADHD patients as compared to healthy controls. Undoubtedly, prospective clinical studies would be needed to confirm the role of these parameters as markers in ADHD; however, retrospective studies to date indicate the possibility of such potential use. These findings, however, may point to the involvement of inflammation in the pathogenesis of ADHD. NLR is a marker of systemic inflammation that links two different immune pathways. Neutrophils are front-line cells of innate immune defense and engage in a variety of physiological and pathological processes, such as inflammation, autoimmunity, and others (31). While migrating to the site of infection, they engage in phagocytosis and subsequently execute the elimination of pathogens (32). On the other hand, lymphocytes play a central role in adaptive immunity by actively contributing to the recognition of pathogen-specific immune responses, the generation of immune memory, and the maintenance of host immune homeostasis (33). The result of our meta-analysis may indicate that innate immunity is probably more responsible than adaptive immunity in the pathogenesis of ADHD. However, these pathways are connected as indicated by the neutrophil’s interaction with adaptive immunity. Furthermore, both neutrophils and lymphocytes can migrate into the brain, taking part in the development and exacerbation of neuroinflammation (20). Several studies have shown that platelets are important components of both innate and adaptive immunity (34). They are a major source of cytokines and pro-inflammatory molecules and have some similarities with neurons, such as the transport, metabolism, and release of several neurotransmitters. Although this effect was not as strong as that of NLR, our study demonstrated that PLR can suggest neuroinflammation in patients with ADHD. In contrast, the combined MLR score of the four studies showed no significant difference between ADHD patients and health control. Monocytes are involved in innate and adaptive immunity, primarily responsible for phagocytosis, antigen presentation, and cytokine production (35). They can also migrate to the brain, where cooperating with microglia, they contribute to neuroinflammation (36). More research is needed in this field to draw more reliable conclusions.

Only one study included patients treated with psychostimulants. No significant difference was determined between patients who received psychopharmacological treatment for ADHD and patients that did not with regard to NLR and PLR. However, this study was an outlier contributing significantly to the heterogeneity in the meta-analysis comparing ADHD patients with healthy controls, suggesting that psychostimulant treatment may affect peripheral inflammatory markers (21). Some previous studies reported proinflammatory properties of psychostimulants. Preclinical study found that chronic treatment with methylphenidate can cause oxidative stress, neuroinflammation and neurodegeneration in hippocampus of adult rats (37). Moreover, young animals appear to be more susceptible to damage induced by methylphenidate (38). Another animal study found that amphetamine-like agents induced neuroinflammation in the hippocampus of mice, which could be prevented by anti-inflammatory treatment (39). However, in human study lower levels of IFN-γ and IL-13 in treated patients with ADHD compared to untreated patients were reported, indicating an effect of medication on levels of these cytokines (11). Further prospective studies are needed to assess the correlation of NLR, MLR, PLR, and others inflammatory markers with drug treatment in ADHD.

Our study did not find any significant association between NLR, PLR, and MLR, and specific subtypes of ADHD diagnosis. These result suggest that increased inflammation may be a feature of overall ADHD diagnosis rather than specific subtypes. Our finding are consistent with those of other studies on inflammatory markers in ADHD. No difference in Il-6, TNF-α and morning cortisol measurement was observed between combined and inattentive ADHD subtypes (40, 41). Additionally, inflammation may indicate a ADHD diagnosis, but not reflect the severity of the disease. None of the studies included in the review confirmed a correlation between scores on the clinical assessment scale and inflammatory markers.

As regards the relationship between inflammatory markers and ADHD symptoms, this issue remains unclear. In study conducted by Oates et al., elevated levels of IL-13 were associated with inattention, while elevated levels of IL-16 were associated with hyperactivity. Oppositional symptoms were found to be associated with a decrease in IL-2. Furthermore, an increase in IL-16 and a decrease in TNF-α and IL-6 were positively correlated with commissions errors (10). In our review, two studies found no correlation with scores on the T-DSM-IV-S scale (21, 28), while one observed a tendency to correlate with conduct disorder symptoms (23). Interestingly, PLR was correlated with hyperactive/impulsive scores assessed by parents and hyperactive/impulsive scores, oppositional behaviors and conduct disorder symptoms assessed by teachers (30). Although the results obtained so far do not appear to support a correlation between symptoms and NLR, MLR, and PLR, more research is needed to evaluate this issue.

Current treatment of ADHD involves symptom reduction and is purely symptomatic. Investigating the role of inflammation in the pathogenesis of ADHD may contribute to the development of new therapies. To date, no study has been conducted to assess the efficacy of anti-inflammatory treatment in ADHD, with used e.g., non-steroidal anti-inflammatory drugs (NSAIDs). Clinical trials have confirmed the efficacy and safety of NSAIDs in other mental disorders in which inflammation plays a role in the pathogenesis, such as mood disorders (42, 43). However, there are studies evaluating additional treatment of nutraceuticals with postulated anti-inflammatory properties in ADHD. A randomized controlled trial conducted over 8 weeks with 103 children showed that supplementation with omega-3 fatty acids, which are involved in arachidonic acid pathways, led to a significant reduction in inflammatory markers such as CRP and IL-6. In addition, this supplementation resulted in a noticeable clinical improvement (44). A recent meta-analysis showed that polyunsaturated fatty acids (PUFA) may have a limited effect on treatment outcomes in ADHD (45). Nevertheless, a better understanding of the inflammatory background in ADHD may help to identify patients with higher levels of inflammation. For these patients, the probability of response to add-on anti-inflammatory treatment could be much greater and result in an improved quality of life.

In recent years, the number of adults diagnosed with ADHD has been increasing (46). To date, there has been only one study evaluating NLR, MLR, and PLR values in adult patients (47). There were no differences in these parameters compared to the control group, as well as no associations with treatment or clinical symptoms were observed. However, further studies with a prospective, longitudinal model including a larger study group are required for conclusive results.

Our study has certain limitations, that should be acknowledged. Firstly, the correlations presented may not demonstrate a causal relationship. Elevations in inflammatory markers may indicate the role of inflammation in the pathogenesis of ADHD; however, it is also possible that ADHD develops independently and that its particular features, such as hyperactivity, lower social functioning status, or hygiene, contribute to the increase of these markers. Therefore, careful interpretation of the results is required to understand the complex association between ADHD and inflammatory markers. Secondly, with the exception of one study, all the included studies were conducted within the same country. A recent meta-analysis demonstrated that individuals with depression in Turkey and China displayed significantly higher NLR values compared to other countries. Conversely, PLR was higher among individuals from China rather than Turkey (17). This indicates that these parameters may vary depending on ethnicity. Thirdly, only one study included pharmacologically treated patients. Additional investigations are needed to evaluate whether these markers can serve as indicators of treatment efficacy. Fourthly, we did not conduct a subgroup analysis based on gender differences, as the majority of the studies did not include gender-specific groups. Gender disparities may play a crucial role and potentially explain the diverse range of outcomes observed. Fifth, inflammatory markers may be affected by many other variables, such as medical disease, nutrition, or smoking, which many studies have not reported. Future studies should consider these variables in the evaluation of NLR, MLR, and PLR.

## Data availability statement

The original contributions presented in this study are included in this article/supplementary material, further inquiries can be directed to the corresponding author.

## Author contributions

AG: Conceptualization, Data curation, Formal analysis, Investigation, Methodology, Software, Writing – original draft, Writing – review and editing. SM: Data curation, Investigation, Writing – original draft. MG: Data curation, Investigation, Writing – original draft. AA: Funding acquisition, Supervision, Writing – review and editing. PM: Project administration, Supervision, Writing – review and editing. MD: Funding acquisition, Project administration, Supervision, Writing – review and editing.

## References

[1] Posner J Polanczyk GV Sonuga-Barke E. Attention-deficit hyperactivity disorder. *Lancet.* (2020) 395:450–62. 10.1016/S0140-6736(19)33004-1 31982036 PMC7880081

[2] Harpin V. The effect of ADHD on the life of an individual, their family, and community from preschool to adult life. *Arch Dis Child.* (2005) 90 (Suppl. 1):i2–7. 10.1136/adc.2004.059006 15665153 PMC1765272

[3] Sharma A Couture J. A review of the pathophysiology, etiology, and treatment of attention-deficit hyperactivity disorder (ADHD). *Ann Pharmacother.* (2014) 48:209–25. 10.1177/1060028013510699 24259638

[4] Saccaro L Schilliger Z Perroud N Piguet C. Inflammation, anxiety, and stress in attention-deficit/hyperactivity disorder. *Biomedicines.* (2021) 9:1313. 10.3390/biomedicines9101313 34680430 PMC8533349

[5] Dunn G Nigg J Sullivan E. Neuroinflammation as a risk factor for attention deficit hyperactivity disorder. *Pharmacol Biochem Behav.* (2019) 182:22–34. 31103523 10.1016/j.pbb.2019.05.005 PMC6855401

[6] Instanes J Halmøy A Engeland A Haavik J Furu K Klungsøyr K. Attention-deficit/hyperactivity disorder in offspring of mothers with inflammatory and immune system diseases. *Biol Psychiatry.* (2017) 81:452–9. 10.1016/j.biopsych.2015.11.024 26809250

[7] Lin Y Chen Y Gau S Yeh T Fan H Hwang Y Associations between allergic diseases and attention deficit hyperactivity/oppositional defiant disorders in children. *Pediatr Res.* (2016) 80:480–5. 10.1038/pr.2016.111 27356086

[8] Darwish A Elgohary T Nosair N. Serum Interleukin-6 level in children with Attention-Deficit Hyperactivity Disorder (ADHD). *J Child Neurol.* (2019) 34:61–7. 10.1177/0883073818809831 30430896

[9] Donfrancesco R Nativio P Borrelli E Giua E Andriola E Villa MP Serum cytokines in pediatric neuropsychiatric syndromes: focus on Attention deficit hyperactivity disorder. *Minerva Pediatr.* (2021) 73:398–404. 10.23736/S2724-5276.16.04642-9 28006890

[10] Oades RD Myint A-M Dauvermann MR Schimmelmann BG Schwarz MJ. Attention-deficit hyperactivity disorder (ADHD) and glial integrity: an exploration of associations of cytokines and kynurenine metabolites with symptoms and attention. *Behav Brain Funct.* (2010) 6:32. 10.1186/1744-9081-6-32 20534153 PMC2900218

[11] Oades RD Dauvermann MR Schimmelmann BG Schwarz MJ Myint A-M. Attention-deficit hyperactivity disorder (ADHD) and glial integrity: S100B, cytokines and kynurenine metabolism–effects of medication. *Behav Brain Funct.* (2010) 6:29. 10.1186/1744-9081-6-29 20509936 PMC2889842

[12] Urbanowicz T Olasińska-Wiśniewska A Michalak M Rodzki M Witkowska A Straburzyńska-Migaj E The prognostic significance of neutrophil to Lymphocyte Ratio (NLR), Monocyte to Lymphocyte Ratio (MLR) and Platelet to Lymphocyte Ratio (PLR) on Long-Term Survival in Off-Pump Coronary Artery Bypass Grafting (OPCAB) procedures. *Biology.* (2021) 11:34. 10.3390/biology11010034 35053032 PMC8772913

[13] Mihai A Caruntu A Opris-Belinski D Jurcut C Dima A Caruntu C The predictive role of Neutrophil-to-Lymphocyte Ratio (NLR), Platelet-to-Lymphocyte Ratio (PLR), Monocytes-to-Lymphocyte Ratio (MLR) and gammaglobulins for the development of cutaneous vasculitis lesions in Primary Sjögren’s syndrome. *J Clin Med.* (2022) 11:5525. 10.3390/jcm11195525 36233393 PMC9572220

[14] Qun S Tang Y Sun J Liu Z Wu J Zhang J Neutrophil-to-lymphocyte ratio predicts 3-month outcome of acute ischemic stroke. *Neurotox Res.* (2017) 31:444–52. 10.1007/s12640-017-9707-z 28181171

[15] Erre GL Paliogiannis P Castagna F Mangoni AA Carru C Passiu G Meta-analysis of neutrophil-to-lymphocyte and platelet-to-lymphocyte ratio in rheumatoid arthritis. *Eur J Clin Invest.* (2019) 49:e13037. 10.1111/eci.13037 30316204

[16] Zhao W-M Tao S-M Liu G-L. Neutrophil-to-lymphocyte ratio in relation to the risk of all-cause mortality and cardiovascular events in patients with chronic kidney disease: a systematic review and meta-analysis. *Ren Fail.* (2020) 42:1059–66. 10.1080/0886022X.2020.1832521 33081569 PMC7668415

[17] Su M Ouyang X Song Y. Neutrophil to lymphocyte ratio, platelet to lymphocyte ratio, and monocyte to lymphocyte ratio in depression: {A} meta-analysis. *J Affect Disord.* (2022) 308:375–83. 10.1016/j.jad.2022.04.038 35439466

[18] Mazza MG Lucchi S Tringali AGM Rossetti A Botti ER Clerici M. Neutrophil/lymphocyte ratio and platelet/lymphocyte ratio in mood disorders: a meta-analysis. *Prog Neuropsychopharmacol Biol Psychiatry.* (2018) 84:229–36. 10.1016/j.pnpbp.2018.03.012 29535038

[19] Mazza MG Lucchi S Rossetti A Clerici M. Neutrophil-lymphocyte ratio, monocyte-lymphocyte ratio and platelet-lymphocyte ratio in non-affective psychosis: a meta-analysis and systematic review. *World J Biol Psychiatry.* (2020) 21:326–38. 10.1080/15622975.2019.1583371 30806142

[20] Cheng Y Wang Y Wang X Jiang Z Zhu L Fang S. Neutrophil-to-lymphocyte ratio, platelet-to-lymphocyte ratio, and monocyte-to-lymphocyte ratio in depression: an updated systematic review and meta-analysis. *Front Psychiatry.* (2022) 13:893097. 10.3389/fpsyt.2022.893097 35782448 PMC9240476

[21] Onder A Coban OG Adanir AS Önder A Gizli Çoban Ö Sürer Adanır A. Elevated neutrophil-to-lymphocyte ratio in children and adolescents with attention-deficit/hyperactivity disorder. *Int J Psychiatry Clin Pract.* (2021) 25:43–8. 10.1080/13651501.2020.1804940 32787596

[22] Akinci MA Uzun N Akıncı MA Uzun N. Evaluation of hematological inflammatory markers in children and adolescents with attention deficit/hyperactivity disorder. *Bratislava Med J.* (2021) 122:256–62. 10.4149/BLL_2021_042 33729818

[23] Topal Z Tufan AE Karadag M Gokcen C Akkaya C Sarp AS Evaluation of peripheral inflammatory markers, serum B12, folate, ferritin levels and clinical correlations in children with autism spectrum disorder (ASD) and attention deficit hyperactivity disorder (ADHD). *Nord J Psychiatry.* (2022) 76:150–7. 10.1080/08039488.2021.1946712 34232109

[24] Aksu GG Dağ P. Evaluation of the indicators of inflammation in children and adolescents with attention deficit and hyperactivity disorder: effect of sex and subtype. *Duzce Med J.* (2020) 22:84–90. 10.18678/dtfd.690128

[25] Page MJ Moher D Bossuyt PM Boutron I Hoffmann TC Mulrow CD PRISMA 2020 explanation and elaboration: updated guidance and exemplars for reporting systematic reviews. *BMJ.* (2021) 372:n160. 10.1136/bmj.n160 33781993 PMC8005925

[26] Wells GA Shea B O’Connell D Peterson J Welch V Losos M *The Newcastle-Ottawa Scale (NOS) for Assessing the Quality of Nonrandomised Studies in Meta-Analyses.* (2000). Available online at: https://www.ohri.ca/programs/clinical_epidemiology/oxford.asp (accessed June 1, 2023).

[27] Fahiem RA Mekkawy LH. A new perspective of attention deficit hyperactivity disorder associated with delayed language development: an Egyptian sample. *Psychiatry Investig.* (2022) 19:164–70. 10.30773/pi.2021.0232 35196826 PMC8958202

[28] Avcil S. Evaluation of the neutrophil/lymphocyte ratio, platelet/lymphocyte ratio, and mean platelet volume as inflammatory markers in children with attention-deficit hyperactivity disorder. *Psychiatry Clin Neurosci.* (2018) 72:522–30. 10.1111/pcn.12659 29607599

[29] Alpay M Yektas C Karacor K. The systemic cell apoptotic-based neutrophil-lymphocyte ratio: experience in children diagnosed with ADHD and Autism Spectrum Disorder. *Konuralp Tip Derg.* (2021) 13:74–81. 10.18521/ktd.737628

[30] Yektas C Tufan AE Kilicaslan O Yazici M Karakaya SEK Sarigedik E. Elevated monocyte levels maybe a common peripheral inflammatory marker in specific learning disorders and attention deficit/hyperactivity disorder. *Psychiatry Behav Sci.* (2022) 12:125–33. 10.5455/PBS.20210518080022

[31] Liew PX Kubes P. The neutrophil’s role during health and disease. *Physiol Rev.* (2019) 99:1223–48. 10.1152/physrev.00012.2018 30758246

[32] Mayadas TN Cullere X Lowell CA. The multifaceted functions of neutrophils. *Annu Rev Pathol.* (2014) 9:181–218. 10.1146/annurev-pathol-020712-164023 24050624 PMC4277181

[33] Bonilla FC Oettgen HC. Adaptive immunity. *J Allergy Clin Immunol.* (2010) 125:S33–40. 10.1016/j.jaci.2009.09.017 20061006

[34] Koupenova M Clancy L Corkrey HA Freedman JE. Circulating platelets as mediators of immunity, inflammation, and thrombosis. *Circ Res.* (2018) 122:337–51. 10.1161/CIRCRESAHA.117.310795 29348254 PMC5777300

[35] Germic N Frangez Z Yousefi S Simon H-U. Regulation of the innate immune system by autophagy: monocytes, macrophages, dendritic cells and antigen presentation. *Cell Death Differ.* (2019) 26:715–27. 10.1038/s41418-019-0297-6 30737475 PMC6460400

[36] Wohleb ES Fenn AM Pacenta AM Powell ND Sheridan JF Godbout JP. Peripheral innate immune challenge exaggerated microglia activation, increased the number of inflammatory CNS macrophages, and prolonged social withdrawal in socially defeated mice. *Psychoneuroendocrinology.* (2012) 37:1491–505. 10.1016/j.psyneuen.2012.02.003 22386198 PMC3368999

[37] Motaghinejad M Motevalian M Shabab B. Effects of chronic treatment with methylphenidate on oxidative stress and inflammation in hippocampus of adult rats. *Neurosci Lett.* (2016) 619:106–13. 10.1016/j.neulet.2015.12.015 26687276

[38] Foschiera LN Schmitz F Wyse ATS. Evidence of methylphenidate effect on mitochondria, redox homeostasis, and inflammatory aspects: insights from animal studies. *Prog Neuropsychopharmacol Biol Psychiatry.* (2022) 116:110518. 10.1016/j.pnpbp.2022.110518 35092763

[39] Gonçalves J Baptista S Martins T Milhazes N Borges F Ribeiro CF Methamphetamine-induced neuroinflammation and neuronal dysfunction in the mice hippocampus: preventive effect of indomethacin. *Eur J Neurosci.* (2010) 31:315–26. 10.1111/j.1460-9568.2009.07059.x 20074221

[40] Corominas-Roso M Armario A Palomar G Corrales M Carrasco J Richarte V IL-6 and TNF-α in unmedicated adults with ADHD: relationship to cortisol awakening response. *Psychoneuroendocrinology.* (2017) 79:67–73. 10.1016/j.psyneuen.2017.02.017 28262601

[41] Ramos-Quiroga JA Corominas-Roso M Palomar G Ferrer R Valero S Corrales M Cortisol awakening response in adults with attention deficit hyperactivity disorder: subtype differences and association with the emotional lability. *Eur Neuropsychopharmacol.* (2016) 26:1140–9. 10.1016/j.euroneuro.2016.03.014 27084305

[42] Gędek A Szular Z Antosik AZ Mierzejewski P Dominiak M. Celecoxib for mood disorders: a systematic review and meta-analysis of randomized controlled trials. *J Clin Med.* (2023) 12:3497. 10.3390/jcm12103497 37240605 PMC10218898

[43] Dominiak M Gędek A Sikorska M Mierzejewski P Wojnar M Antosik-Wójcińska A. Acetylsalicylic acid and mood disorders: a systematic review. *Pharmaceuticals.* (2022) 16:67. 10.3390/ph16010067 36678565 PMC9861965

[44] Hariri M Djazayery A Djalali M Saedisomeolia A Rahimi A Abdolahian E. Effect of n-3 supplementation on hyperactivity, oxidative stress and inflammatory mediators in children with attention-deficit-hyperactivity disorder. *Malays J Nutr.* (2012) 18:329–35. 24568073

[45] Gillies D Leach MJ Perez Algorta G. Polyunsaturated fatty acids (PUFA) for attention deficit hyperactivity disorder (ADHD) in children and adolescents. *Cochr Datab Syst Rev.* (2023) 4:CD007986. 10.1002/14651858.CD007986.pub3 37058600 PMC10103546

[46] Adamis D Flynn C Wrigley M Gavin B McNicholas F. ADHD in adults: a systematic review and meta-analysis of prevalence studies in outpatient psychiatric clinics. *J Atten Disord.* (2022) 26:1523–34. 10.1177/10870547221085503 35373645

[47] Ceyhun HA Gürbüzer N. New hematological parameters as inflammatory biomarkers: systemic immune inflammation index, platerethritis, and platelet distribution width in patients with adult attention deficit hyperactivity disorder. *Adv Neurodev Disord.* (2022) 6:211–23. 10.1007/s41252-022-00258-6 35573104 PMC9091147

